# Analysis of Trend and Associated Factors of Neuropsychological Development of Infants and Toddlers Based on Longitudinal Data

**DOI:** 10.3390/children8100866

**Published:** 2021-09-28

**Authors:** Deng Chen, Yunzhe Huang, Andrew Swain, Xiaoguang Yang, Jinming Yu

**Affiliations:** 1 School of Public Health, Fudan University, Shanghai 200433, China; 19111020021@fudan.edu.cn; 2 Shanghai VIP Health Care Co., Ltd. (C-LAP), Shanghai 200025, China; jay.huang@c-lap.com (Y.H.); andrewswain@c-lap.com (A.S.); 3 China Hospital Development Institute, Shanghai Jiao Tong University, Shanghai 200032, China; yangxg@fudan.edu.cn

**Keywords:** trend, associated factors, neuropsychological development, infants and toddlers, longitudinal data

## Abstract

Objective: To explore the trend and associated factors of neuropsychological development of infants and toddlers in China. Methods: A longitudinal study was conducted among 619 infants and toddlers (2914 person-times) aged 0 to 36 months from different provinces or cities in China from January 2013 to December 2019. Results: The development age of each area increased with the extension of follow-up time, but this upward trend slowed down with physiological age at first measurement increasing. Among a low age group and each area, most of the development qualification rates in different follow-up periods were higher than that in the baseline (*p* < 0.05); however, many of them were not higher than that in the baseline among the medium or high age group (*p* > 0.05). For the areas of gross motor and self-care, the growth of qualification rate with the extension of follow-up was not obvious in the medium and high age group (both *p* trend > 0.05). Some impact factors of development in all areas were identified. Conclusions: The neuropsychological development delay of various areas of infants and toddlers, especially that of gross motor and self-care, should be paid early (within 1 years old) and constant attention. The impact of gender and maternal age on the development of young children has been further confirmed in the present study.

## 1. Introduction

Previous studies have found that the neuropsychological development status of infants in China for the past few years is not as optimistic as expected. Some development disability among sports, social adjustment and mental area were observed in a survey of young children from Jiangsu province, and the largest delay ratio was in sports [1]. A study of 3080 children aged 4 to 30 months in Daxing District, Beijing from 2016 to 2018 indicated that boys and girls exhibit different amounts of susceptibility to developmental problems of language, fine movement and social behavior, and the delay of the former was more distinct [2].

Zero to three years old is a critical period for the growth and development of infants and toddlers. Studies have shown that effective interventions exist for some identified risks, although further research is needed to increase our ability to promote early child development in low-income and middle-income countries [3].

Not insignificant, furthermore, is the close relationship between the neuropsychological development of different areas in early childhood and some of the performance into adolescence and adulthood. Studies provide evidence that children with strong fine motor skills measured with a composite demonstrated better mathematics performance at kindergarten entry and make greater gains of mathematics, reading and writing over the year [4,5,6]. Gross motor delay is considered to be an important precursor of developmental disorders and nervous system related diseases, and effective assessment of the development of the gross motor is the key to early recognition of anomalies [7]. Some other findings suggest that processing speed and early language skills are fundamental to intellectual functioning, and that language development is guided by learning and representational principles shared across cognitive and linguistic domains [8]. Moreover, the infant-–mother attachment data can significantly enhance the prediction of an advanced understanding of mixed emotions at six years [9].

However, when taking account of the rate of neuropsychological development, there may be great gaps between different areas. Genes are generally credited with causing this imbalance, and the study of genetic disorders that affect neurodevelopment has led to a rich body of interdisciplinary research in genetics, neuroscience and psychology [10]. Culture or region may also be associated with the difference of the rate of neuropsychological development of different areas. A prior study applying conventional clustering techniques to classify the experimental data found a stable representation of two clusters of children with distinctive traits, with cultural factors contributing to this classification scheme [11].

In addition, neuropsychological development of infants and young children is associated with many other factors. In a longitudinal study, girls had better fine motor and language skills than boys at 2 and 3 years of age; however, at 5–6 years of age, differences in fine motor skills were of a smaller magnitude and those in language skills were no longer significant [12]. These findings are consistent with the idea that sex differences in biological factors may play a substantial role in the greater verbal and fine motor abilities observed in girls during the preschool period [12]. Another study find that Autism spectrum disorders (ASD) is significantly associated with increased paternal age, schizophrenia (SCZ) has significant associations for fathers aged > 35 years, and for major depressive disorder (MDD), both younger and older fathers have increased odds [13]. Results from a population-based cohort study indicated that increasing maternal age was associated with a lesser risk of developmental vulnerability for children born to mothers aged 15 years to about 30 years. In contrast, increasing maternal age beyond 35 years was generally associated with increasing vulnerability, broadly equivalent to the risk for children born to mothers in their early twenties, which is highly relevant in the international context of later childbearing [14]. Parents’ educational background may also contribute to the performance of different aspects of their young children [15].

Yet, association of gender, region, and parents’ age and education with neuropsychological development of young children may need to be tested and confirmed by further study of longer-term, larger scale or sample size. Therefore, in this paper, through a retrospective longitudinal data of different regions in China from 2013 to 2019, we explored trends and associated factors of development in gross motor, fine motor, cognition, language, social emotion and self-care of infants and toddlers. Insight into these trends and associated factors will help us to find and intervene in target groups in time and improve their status of physiological and psychological health.

## 2. Materials and Methods

### 2.1. Participants

This study was carried out at Shanghai VIP Health Care Co., Ltd. (C-LAP) in Shanghai China, and we collected the longitudinal testing data of neuropsychological development of the infants and toddlers aged 0 to 36 months in Beijing, Shanghai, Guangdong and other provinces or cities from January 2013 to December 2019. A total of 645 cases (3088 person-times) were originally included in our study, and 26 of them were deleted due to the lack of information; hence, a total of 619 cases (2914 person-times) were selected eventually. Figure S1 shows the flowchart of case selection. The written informed consent to participate was obtained from the parent or legal guardian of the children. Each participant was provided with some parenting advices. This study was approved by the Institutional Review Board of the School of Public Health, Fudan University (IRB00002408, FWA00002399; approval number IRB#2019-04-0741).

### 2.2. Measurement

The development of different areas was evaluated by the Chinese Learning Accomplishment Profile (C-LAP), which is a criterion-referenced assessment that provides a systematic method for observing and assessing the individual skill development of children functioning in the 0–36 age range. In the presurvey, the evaluation system had good reliability and validity, in which the internal consistency was supported by Cronbach’s α coefficient of 0.95. The outcomes are presented by developmental age, taking the physiological age as a reference. On the one hand, the change of development in a certain area can be observed vertically, and on the other, the synchronicity of the development between different areas can be compared.

The context of the instrument is arranged from easy to difficult, and only children that pass the former questions are able to answer the latter ones. The first item corresponding to the physiological month age was selected as the starting point of the test; if a child passes it, then the next item will be tested, and if not, the researcher goes back 8 items from it and reaches a new starting point, and so on.

### 2.3. Development Rating Based on the Norms

The development rating (“Qualified” or “Delayed”) of each area of infants and toddlers was based on the norm population in low age group (0–12 months, 965 cases), medium age group (12–24 months, 870 cases) and high age group (24–36 months, 1004 cases), which shared similar demographic characteristics with our study population (Table S1). The inclusion criteria of the norm population were as following: (a) 0–3 years old; (b) Routine physical examination was normal; (c) Without development disorders or diseases diagnosed such as cerebral palsy (CP), autism spectrum disorder (ASD), Down syndrome (DS), etc.

The difference between development age and physiological age was adopted as the rating reference, and the lower limit of the 95% reference range was taken as the norm standard since poorer development was considered abnormal. Table S2 showed the rating criteria based on norms of three age groups.

### 2.4. Statistical Analysis

Mean and standard error were calculated for continuous variables, and numbers and percentages were computed for categorical variables. The analysis of variance and chi-square test were used to compare, respectively, the mean age and qualification rates of development of different areas in the same follow-up time and age group at the first measurement. Generalized linear mixed effect model was used for multivariate correction, and linear mixed effect models were performed to explore the associated factors of the neuropsychological development of the young children, in that they can well control the influence of individual self-correlation in longitudinal data and detect the interaction between independent variables.

Since the sample was sufficient (more than 20 times the number of variables), the possible associated variables of “Gender”, “Province”, “Father’s education”, “Mother’s education”, “Physiological age at first measurement”, “Paternal age” and “Maternal age” were all included as the independent variables, without performing the univariate analysis.

SAS 9.4 and R 3.6.2 statistical software packages were used for data analysis and plotting. A two-sided *p* value < 0.05 was considered as the significant level.

## 3. Results

### 3.1. Demographics

The basic demographic information, including gender, month of age at first test, province, parental education and age are presented in Table 1. Among the 619 cases (345 males, 274 female), the proportion of boys is slightly higher than that of girls, which is roughly in line with the male-to-female ratio of the total population of infants and toddlers in China. At the first test, young children with a physiological age of more than 12 months old account for the highest proportion, up to 74%. Shanghai is the main place where the children come from, accounting for nearly 50%. In general, the distribution of educational background of both parents is approximately equal, among which, the proportion of father’s postgraduate education is slightly higher than that of mother, while for the proportion of undergraduate or junior college education, the opposite is true. The paternal age is generally higher, and the proportion of fathers in all childbearing age groups above 30 years old is higher than that of mothers.

### 3.2. Mean and Variation Trend of Development Age

Table 2 shown the mean developmental ages and their standard errors of infants and toddlers with physiological month in low, medium and high age group measured for the first time in different areas and follow-up periods (baseline, 1–3 months, 4–6 months and ≥7 months). Mean development age of follow-up periods was higher than that of the baseline in all areas and follow-up periods (all *p* < 0.001); and as the extension of follow-up time of each area in all physiological age groups at first measurement, the mean value of the developing age also showed an increasing trend (all *p* trend < 0.001). However, for the objects in the high age group at first measurement of each area, the mean differences of the developmental age between “≥7 months” and “4–6 months” were far lower than that between “4–6 months“ and “1–3 months”.

**Figure 1:**
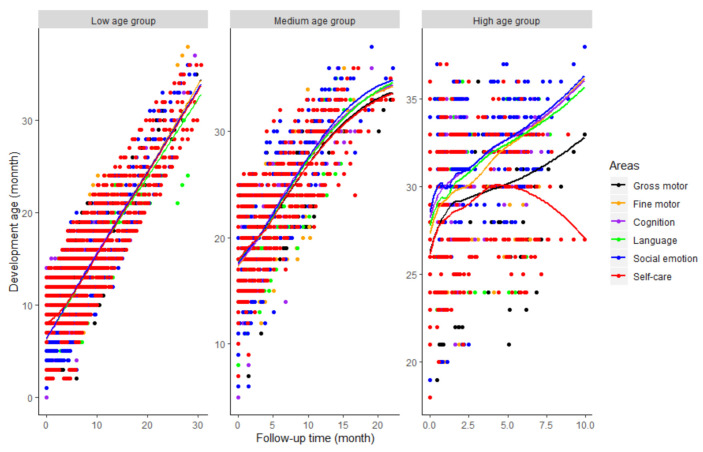
Development Trend of different areas with follow-up time in different age groups at first measurement.

Figure 1 also reflected this trend: that was, in the low age group, the development age of each area increased almost linearly with the extension of follow-up time; in the middle, this upward trend slowed down later; and in the high, the downward trend of the growth rate was more obvious, especially for the area of gross motor and self-care.

### 3.3. Development Qualification Rate and the Impact of Follow-Up Time

The development qualification rate of infants and toddlers during each follow-up period in all age groups and areas was presented in Table 3. Among low age group and each area, most of the development qualification rates in different follow-up periods were higher than that in the baseline (*p* < 0.05); however, many of them were not higher than that in the baseline among the medium or high age group, especially for the areas of gross motor and self-care and the follow-up of >7 months (*p* > 0.05). The development qualification rate of all areas showed a significant increasing trend with the extension of follow-up time in low age group, while only that of cognition and language in medium age group and cognition and social emotion in high age group showed a similar trend (all *p* trend < 0.05). For the areas of gross motor and social emotion, it should be noted, the growth of qualification rate with the extension of follow-up was not obvious in the medium and high age group (both *p* trend > 0.05).

**Figure 2:**
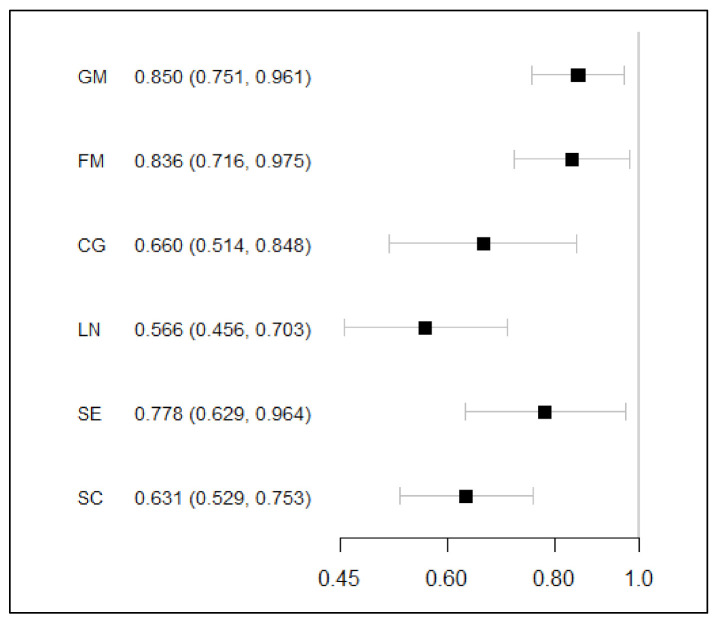
Impact of “Follow-up time” on development rating in six areas. Note: Generalized linear mixed effect model was adopted to control the influence of individual self-correlation and adjust the variables of “Gender”, “Province”, “Father’s education”, “Mother’s education”, “Physiological age at first measurement”, “Paternal age” and “Maternal age”; GM = Gross motor, FM = Fine motor, CG = Cognition, LG = Language, SE = Social emotion, SC = Self-care.

After adjusting the variables “Gender”, “Province”, “Father’s education”, “Mother’s education”, “Physiological age at first measurement”, “Paternal age” and “Maternal age”, “Follow-up time” turned out to be the independent protective factor of qualified development of all areas (*p* < 0.05), with odds ratio (OR) from 0.566 (95% CI: 0.456–0.703) to 0.850 (95% CI: 0.751–0.961) (Figure 2).

### 3.4. Analysis of Associated Factors of Development in Each Area

“Gender”, “Province”, “Father’s education”, “Mother’s education”, “Physiological age at first measurement”, “Paternal age” and “Maternal age” were included in the linear mixed effect model. Table 4 presents the analysis results of development associated factors of each area based on the linear mixed effect model. It reflects that the common independent factors influencing the development of the six areas within 0–36 months were “Province” and “Physiological age at first measurement”. The development age of each area increased with the physiological age at the first measurement. Taking children from Beijing as the reference, children from Shanghai and Guangdong were of relative underperformance and outperformance, respectively. In addition, the development of girls was better than that of boys in the four areas of fine motor, language, cognition and self-care (especially in self-care), and maternal age was a risk factor for the development of the four areas of gross motor, cognition, language and social emotion.

## 4. Discussion

To our knowledge, this paper is the first to comprehensively explore the trend and associated factors of neuropsychological development of different areas over time of infants and toddlers aged 0–3 years old through the analysis of longitudinal observation data, filling in the gaps in relevant research fields in China.

Our study indicates that with the increase of the physiological age at the first measurement, the developmental age of each area rose rapidly and then slowly with time. Furthermore, we can draw the conclusion that a similar development trend was shared by fine motor, cognition, language, and social emotion, and by gross motor and self-care, respectively. This is in line with some studies’ conclusion suggesting that fine motor skills rather than gross motor skills predict improvements in preschoolers’ cognitive and social skills [16,17]. Previous studies also suggested a positive statistically significant relationship between the children’s gross motor skills and self-care skills [18,19], which might be attributed to the fact that many self-care implementations are based primarily on gross motor. For example, touch processing difficulties may affect grooming, bathing and dressing [20].

The qualification rate calculated by Chinese norms indicated almost upward trend with follow-up time in all areas, even adjusting the important variables such as “Physiological age at first measurement”. Nevertheless, the growth trend of qualification rate of most areas (especially for gross motor and self-care) was not obvious in medium or high age groups compared with that in low age group. These indicate that all areas, especially gross motor and self-care, should be paid timely and constant attention in the first two years of young children. If developmental delay occurs during that period, the effect of prompt intervention turns out to be obvious [4]; but once developmental problems are not found until the ages of 2 to 3 years, the improvement from intervention might be limited [21].

Consistent with previous studies, we found that girls outperformed boys in areas of fine motor skills, language, cognition and self-care [12,22]. However, using multivariate regression, our study indicated that gender was not significantly associated with development of gross motor or social emotion. Previous studies have also shown that gender was not an independent factor influencing development of gross motor [23], while preschool girls developed better social emotions than boys [24]. We could not conclude that the developmental quotient (DQ) of male infants is lower than that of female infants, but the phenomenon of male underdevelopment was prominent [2], and the specific mechanism of which deserves further study [25].

The results in this study indicated a regional or cultural difference in the development of gross motor, fine motor, cognition, language, social skills and self-care of infants and toddlers, which resembled that in the earlier researches [11,26]. In addition, development of infants and toddlers from different provinces varied widely in this study. Children from Guangdong province seemed to perform better than those from Beijing and Shanghai in all areas, which indeed reflected certain problems of development imbalance regionally. Yet, it ought to be noted the selection bias in our study, in that the observation objects from Shanghai account for nearly 50%; thus, we still need to make the conclusions with caution.

Educational background of fathers and mothers had statistical sense in the univariate regression, even though it showed no statistical significance in multivariate regression. Our study indicated that the father’s education tended to be inversely correlated with the development of all areas except for gross and fine motor, while the mother’s education was positively correlated with that of almost all areas. This is not completely consistent with the previous study on the positive correlation between parental education and development of infants and toddlers [15,27]. The possible reason may lie in that fathers with higher education spent less time with their children, which caused a negative impact on the neuropsychological development of the young children [27].

Our study found that maternal age is a risk factor for development of all areas of young children, which agrees well with the conclusions from prior studies that older mothers might be at higher risk to have children with autism spectrum disorders and bipolar disorders [28,29]. It is also consistent with another study in which the risk of neuropsychological developmental vulnerability in young children increased with the childbearing age of mothers who were older than 35 years old [14]. However, it represents a departure from the existing literature, that is, after adjusting for paternal age, the maternal age has no independent influence on the outcomes of mental health of offspring [30].

Several limitations should be considered in interpreting our results. First, the data are retrospective, and the accumulation process is not strictly controlled, which hinders us from obtaining detail sociodemographic information. Hence, the number of associated variables included currently is limited, and we will add more associated information in future studies to gain a deeper and comprehensive understanding of children’s neuropsychological development. Second, most of the objects came from the relatively developed provinces and cities in China; thus, the popularization of the results needs a further examination. Third, we only observed the development in different areas of 0–3-year-old infants and toddlers, the development of whom over 3 years old is unknown.

## 5. Conclusions

In conclusion, our findings demonstrate the development trend of fine motor, cognition, language, and social emotion is closer, while that of gross motor and self-care is similar. Neuropsychological development of all areas, especially gross motor and self-care, should also be paid attention to within 1 year old and needs constant attention, to detect and correct the potential development problems in a timely way. It is undesirable not to focus on this until the child is over 1 year old. The influence of gender and age on the neuropsychological development of infants and toddlers has been further confirmed in this study, while the regional impact on it needs to be supported by a larger sample size and longer observation time in future studies.

## Figures and Tables

**Table 1:** Basic demographic information.

Variables	Total (*n* = 619)	Times of Measurement		
		2 (*n* = 238)	3~5 (*n* = 219)	≥6 (*n* = 162)
Gender, *n* (%)				
Male	345 (55.7)	124 (52.1)	127 (58.0)	94 (58.0)
Female	274 (44.3)	114 (47.9)	92 (42.0)	68 (42.0)
Province, *n* (%)				
Beijing	164 (26.5)	21 (8.8)	109 (49.8)	34 (21.0)
Shanghai	307 (49.6)	198 (83.2)	77 (35.2)	32 (19.8)
Guangdong	114 (18.4)	9 (3.8)	15 (6.8)	90 (55.6)
Others	34 (5.5)	10 (4.2)	18 (8.2)	6 (3.7)
Father’s education, *n* (%)				
Postgraduate	60 (9.7)	16 (6.7)	27 (12.3)	17 (10.5)
Bachelor	406 (65.6)	136 (57.1)	138 (63)	132 (81.5)
Junior college	106 (17.1)	64 (26.9)	32 (14.6)	10 (6.2)
High school or below	47 (7.6)	22 (9.2)	22 (10)	3 (1.9)
Mother’s education, *n* (%)				
Postgraduate	42 (6.8)	9 (3.8)	19 (8.7)	14 (8.6)
Bachelor	411 (66.4)	148 (62.2)	133 (60.7)	130 (80.2)
Junior college	116 (18.7)	57 (23.9)	44 (20.1)	15 (9.3)
High school or below	50 (8.1)	24 (10.1)	23 (10.5)	3 (1.9)
Physiological age at first measurement, *n* (%)				
Low age group	160 (25.8)	69 (29.0)	45 (20.5)	45 (27.8)
Medium age group	206 (33.3)	119 (50.0)	111 (50.7)	23 (14.2)
High age group	253 (40.9)	50 (21.0)	63 (28.8)	94 (58.0)
Paternal age (yrs), *n* (%)				
<30	185 (29.9)	73 (30.7)	58 (26.5)	54 (33.3)
30–35	283 (45.7)	121 (50.8)	99 (45.2)	63 (38.9)
36–40	113 (18.3)	32 (13.4)	50 (22.8)	31 (19.1)
>40	38 (6.1)	12 (5.0)	12 (5.5)	14 (8.6)
Maternal age (yrs), *n* (%)				
<30	284 (45.9)	73 (30.7)	58 (26.5)	54 (33.3)
30–35	263 (42.5)	121 (50.8)	99 (45.2)	63 (38.9)
36–40	62 (10.0)	32 (13.4)	50 (22.8)	31 (19.1)
>40	10 (1.6)	12 (5.0)	12 (5.5)	14 (8.6)

**Table 2:** Mean age of development of each area in different follow-up time.

Areas	Follow-Up Time (Month)	Physiological Age Group at First Measurement (Month)								
		Low Age Group			Medium Age Group			High Age Group		
		*n*	Mean ± Sd	*p* Trend	*n*	Mean ± Sd	*p* Trend	*n*	Mean ± Sd	*p* Trend
GM	Baseline	190	6.25 ± 3.34	<0.001	157	17.07 ± 3.75	<0.001	167	26.32 ± 3.50	<0.001
	1–3	175	7.81 ± 3.51 ***		145	18.94 ± 3.72 ***		165	28.60 ± 3.44 ***	
	4–6	134	10.27 ± 3.75 ***		58	21.54 ± 3.48 ***		43	30.23 ± 3.34 ***	
	≥7	105	17.77 ± 6.11 ***		44	27.15 ± 4.21 ***		24	30.88 ± 2.84 ***	
FM	Baseline	192	6.23 ± 3.17	<0.001	120	16.97 ± 4.23	<0.001	169	27.30 ± 3.54	<0.001
	1–3	173	7.79 ± 3.39 ***		114	18.93 ± 3.89 ***		165	29.46 ± 3.29 ***	
	4–6	134	10.31 ± 3.61 ***		57	21.85 ± 3.94 ***		44	32.34 ± 2.53 ***	
	≥7	105	17.80 ± 6.17 ***		46	27.64 ± 4.40 ***		24	33.36 ± 2.50 ***	
CG	Baseline	193	6.50 ± 3.20	<0.001	124	17.12 ± 4.20	<0.001	168	28.21 ± 3.31	<0.001
	1–3	173	7.78 ± 3.45 ***		115	18.99 ± 3.92 ***		165	30.25 ± 3.05 ***	
	4–6	134	10.27 ± 3.62 ***		55	21.90 ± 3.96 ***		44	32.77 ± 2.13 ***	
	≥7	105	17.79 ± 6.01 ***		44	27.74 ± 3.91 ***		24	33.36 ± 2.75 ***	
LN	Baseline	206	6.36 ± 3.16	<0.001	156	17.38 ± 4.30	<0.001	251	27.79 ± 3.88	<0.001
	1–3	182	7.88 ± 3.43 ***		144	19.28 ± 4.05 ***		245	29.77 ± 3.59 ***	
	4–6	134	10.07 ± 3.53 ***		58	21.68 ± 3.98 ***		41	32.44 ± 2.45 ***	
	≥7	107	17.48 ± 5.86 ***		47	27.47 ± 4.24 ***		24	32.94 ± 3.17 ***	
SE	Baseline	205	6.42 ± 3.36	<0.001	156	17.26 ± 4.29	<0.001	249	28.58 ± 3.86	<0.001
	1–3	184	7.84 ± 3.56 ***		145	19.11 ± 4.09 ***		246	30.43 ± 3.46 ***	
	4–6	134	10.28 ± 3.75 ***		57	21.93 ± 3.86 ***		44	32.63 ± 2.30 ***	
	≥7	106	17.75 ± 6.10 ***		47	24.84 ± 4.53 ***		24	33.88 ± 2.00 ***	
SC	Baseline	138	8.20 ± 2.51	<0.001	157	17.52 ± 4.08	<0.001	248	26.21 ± 3.67	<0.001
	1–3	117	8.71 ± 3.77 ***		145	19.36 ± 3.85 ***		244	28.23 ± 3.38 ***	
	4–6	83	10.27 ± 4.20 ***		58	21.49 ± 3.51 ***		44	30.05 ± 3.20 ***	
	≥7	65	17.64 ± 6.07 ***		47	27.15 ± 4.05 ***		24	29.30 ± 3.17 ***	

Note: GM = Gross motor, FM = Fine motor, CG = Cognition, LG = Language, SE = Social emotion, SC = Self-care; the asterisk indicated that in a specific area and a physiological age group at first measurement, the difference between the development mean of a follow-up period and that of the baseline was statistically significant. *** denoted *p* < 0.001.

**Table 3:** Development qualification rate of each area in different follow-up time.

Areas	Follow-Up Time (Month)	Physiological Age Group at First Measurement (Month)								
		Low Age Group			Medium Age Group			High Age Group		
		*n*	Qualification Rate (%)	*p* Trend	*n*	Qualification Rate (%)	*p* Trend	*n*	Qualification Rate (%)	*p* Trend
GM	Baseline	190	88.9	<0.001	157	95.5	0.375	167	86.2	0.230
	1–3	175	93.7 **		145	97.9 **		165	89.7 *	
	4–6	134	93.3		58	98.3		43	88.4	
	≥7	105	98.1 **		44	95.7		24	91.7	
FM	Baseline	192	91.7	0.008	120	96.7	0.503	169	89.9	0.057
	1–3	173	94.8 **		114	98.2		165	96.4 ***	
	4–6	134	93.3		57	98.3		44	95.5	
	≥7	105	98.1 *		46	97.8		24	91.7	
CG	Baseline	193	75.1	<0.001	124	91.1	0.012	168	95.2	0.033
	1–3	173	90.2 ***		115	94.8 *		165	98.2	
	4–6	134	82.8		55	92.7		44	100	
	≥7	105	96.2 ***		44	97.7 *		24	95.8	
LN	Baseline	206	83.5	<0.001	156	88.5	<0.001	251	88	0.244
	1–3	182	94.5 ***		144	96.5 ***		245	91 **	
	4–6	134	92.5 **		58	98.3 ***		41	95.1	
	≥7	107	96.3 ***		47	97.9 ***		24	91.7	
SE	Baseline	205	89.3	0.016	156	91.7	0.957	249	86.3	<0.001
	1–3	184	96.7 ***		145	94.5 **		246	91.1 ***	
	4–6	134	91.8		57	93		44	95.5 **	
	≥7	106	96.2 **		47	93.6		24	100	
SC	Baseline	138	81.9	<0.001	157	95.5	0.606	248	77	0.993
	1–3	117	89.7 **		145	97.9		244	84 ***	
	4–6	83	96.4 ***		58	98.3		44	86.4	
	≥7	65	96.9 *		47	95.7		24	70.8 *	

Note: GM = Gross motor, FM = Fine motor, CG = Cognition, LG = Language, SE = Social emotion, SC = Self-care; the asterisk indicated that in a specific area and a physiological age group at first measurement, the difference between the qualification rate of a follow-up period and that of the baseline was statistically significant. * denoted *p* < 0.05, ** denoted *p* < 0.01, *** denoted *p* < 0.001.

**Table 4:** Analysis of factors influencing development of each area based on the linear mixed effect model.

Variables	Areas (β, 95%CI)					
	GM	FM	CG	LN	SE	SC
Gender						
Male	Ref	Ref	Ref	Ref	Ref	Ref
Female	0.38 (−0.20, 0.98)	0.71 (0.13, 1.30)	0.64 (0.08, 1.21)	0.77 (0.26, 129)	0.48 (−0.04, 0.99)	0.90 (0.38, 1.43)
Province						
Beijing	Ref	Ref	Ref	Ref	Ref	Ref
Shanghai	−0.79 (−1.72, 0.12)	−1.00 (−1.92, −0.09)	−1.26 (−2.16, 0.38)	−1.48 (−2.18, −0.78)	−1.65 (−2.35, −0.94)	−0.74 (−1.46, −0.04)
Guangdong	2.55 (1.57, 3.53)	2.44 (1.47, 3.40)	2.23 (1.29, 3.17)	1.96 (1.11, 2.82)	1.93 (1.08, 2.78)	3.09 (2.22, 3.96)
Others	−0.03 (−1.32, 1.42)	0.65 (−0.68, 2.01)	0.68 (−0.63, 2.00)	0.07 (−1.19, 1.36)	0.54 (−0.74, 1.86)	2.44 (1.09, 3.81)
Father’s education						
Postgraduate	Ref	Ref	Ref	Ref	Ref	Ref
Bachelor	0.21 (−1.73, 2.16)	0.56 (−1.37, 2.49)	−0.45 (−2.31, 1.43)	−0.06 (−1.71, 1.60)	−0.17 (−1.84, 1.50)	0.15 (−1.52, 1.84)
Junior college	0.18 (−1.52, 1.89)	0.21 (−1.48, 1.90)	−0.68 (−2.32, 0.96)	−0.13 (−1.54, 1.28)	−0.52 (−1.94, 0.90)	−0.21 (−1.63, 1.21)
High school or below	0.19 (−1.51, 1.89)	0.25 (−1.45, 1.94)	−0.62 (−2.26, 1.03)	−0.23 (−1.61, 1.15)	−0.36 (−1.75, 1.03)	−0.28 (−1.66, 1.10)
Mother’s education						
Postgraduate	Ref	Ref	Ref	Ref	Ref	Ref
Bachelor	1.56 (−0.46, 3.58)	0.83 (−1.19, 2.83)	1.65 (−0.30, 3.59)	1.49 (−0.24, 3.22)	0.94 (−0.80, 2.67)	0.29 (−1.47, 2.03)
Junior college	1.11 (−0.56, 2.80)	0.06 (−1.61, 1.73)	0.89 (−0.72, 2.52)	0.83 (−0.54, 2.20)	0.46 (−0.92, 1.84)	−0.18 (−4.57, 1.20)
High school or below	0.67 (−0.97, 2.32)	−0.68 (−2.32, 0.95)	0.13 (−1.46, 1.72)	−0.06 (−1.40, 1.26)	0.06 (−1.28, 1.39)	−0.29 (−1.64, 1.04)
Physiological age at first measurement	0.78 (0.75, 0.82)	0.82 (0.78, 0.85)	0.84 (0.80, 0.87)	0.81 (0.79, 0.84)	0.83 (0.80, 0.86)	0.73 (0.70, 0.76)
Paternal age	0.08 (−0.12, 0.18)	0.04 (−0.06, 0.14)	0.04 (−0.06, 0.13)	0.05 (−0.04, 0.14)	0.05 (−0.04, 0.14)	0.03 (−0.06, 0.12)
Maternal age	−0.17 (−0.29, −0.04)	−0.12 (−0.24, 0.01)	−0.13 (−0.25, −0.01)	−0.11 (−0.22, −0.01)	−0.16 (−0.27, −0.05)	−0.09 (−0.20, 0.02)

Note: GM = Gross motor, FM = Fine motor, CG = Cognition, LG = Language, SE = Social emotion, SC = Self-care.

## Data Availability

The datasets are available on reasonable request. If anyone wants to get access to the data, please send an email to chend19@fudan.edu.cn.

## Supplementary Materials

The following are available online at https://www.mdpi.com/article/10.3390/children8100866/s1. Figure S1: Flow chart of case selection, Table S1: Demographic comparison between the norms and study population in different age groups, Table S2: Rating criteria based on norms of three age groups.
