# Supplementary material for: Analysis of Trend and Associated Factors of Neuropsychological Development of Infants and Toddlers Based on Longitudinal Data
Source: Children (Basel). 2021 Sep 28;8(10):866. doi: 10.3390/children8100866 (PMC8535091; doi:10.3390/children8100866)
Supplement: Supplementary file 1 [file children-08-00866-s001.zip › children-1375603-supplementary.pdf]

children

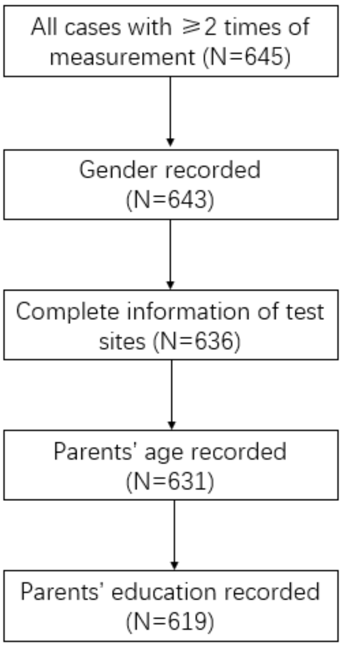

Figure S1. Flow chart of case selection.

Table S1. Demographic comparison between the norms and study population in different age groups.

	Low age group				Medium age group				High age group		
	Norm (*n* = 965)	SP (*n* = 193)	*P* value		Norm (*n* = 870)	SP (*n* = 145)	*P* value		Norm (*n* = 1 004)	SP (*n* = 251)	*P* value
Gender			0.811	Gender			0.817	Gender			0.955
Male	551 (57.1)	112 (58)		Male	471 (54.1)	80 (55.2)		Male	558 (55.6)	140 (55.8)	
Female	414 (42.9)	81 (42)		Female	399 (45.9)	65 (44.8)		Female	446 (44.4)	111 (44.2)	
Province			0.488	Province			0.927	Province			0.168
Beijing	65 (6.7)	12 (6.2)		Beijing	149 (19.4)	26 (17.9)		Beijing	420 (41.8)	120 (47.8)	
Shanghai	562 (58.2)	106 (54.9)		Shanghai	482 (55.4)	83 (57.2)		Shanghai	475 (47.3)	100 (39.8)	
Guangdong	300 (31.1)	63 (32.6)		Guangdong	208 (21.6)	31 (21.4)		Guangdong	64 (6.4)	16 (6.4)	
Others	38 (3.9)	12 (6.2)		Others	31 (3.6)	5 (3.4)		Others	45 (4.5)	15 (6)	
Father's education			0.973	Father's education			0.997	Father's education			0.950
Postgraduate	94 (9.7)	18 (9.3)		Postgraduate	80 (9.2)	14 (9.7)		Postgraduate	99 (9.9)	27 (10.8)	
Bachelor	770 (79.8)	154 (79.8)		Bachelor	579 (66.6)	96 (66.2)		Bachelor	556 (55.4)	135 (53.8)	
Junior college	94 (9.7)	19 (9.8)		Junior college	148 (17)	25 (17.2)		Junior college	221 (22)	55 (21.9)	
High school	7 (0.7)	2 (1)		High school	63 (7.2)	10 (6.9)		High school	128 (12.7)	34 (13.5)	
Mother's education			0.942	Mother's education			0.860	Mother's education			0.963
Postgraduate	65 (6.7)	14 (7.3)		Postgraduate	79 (9.1)	11 (7.6)		Postgraduate	59 (5.9)	15 (6)	
Bachelor	769 (79.7)	151 (78.2)		Bachelor	570 (65.5)	93 (64.1)		Bachelor	595 (59.3)	147 (58.6)	
Junior college	116 (12)	24 (12.4)		Junior college	160 (18.4)	30 (20.7)		Junior college	230 (22.9)	56 (22.3)	
High school	15 (1.6)	4 (2.1)		High school	61 (7)	11 (7.6)		High school	120 (12)	33 (13.1)	
Physiological			0.950	Physiological age (month)			0.979	Physiological age (month)			0.925

age (month)											
0-3	153 (15.9)	31 (16.1)		13-15	200 (23)	34 (23.4)		25-27	119 (11.9)	33 (13.1)	
4-6	154 (16)	33 (17.1)		16-18	132 (15.2)	20 (13.8)		28-30	212 (21.1)	52 (20.7)	
7-9	232 (24)	43 (22.3)		19-21	134 (15.4)	23 (15.9)		31-33	287 (28.6)	68 (27.1)	
10-12	426 (44.1)	86 (44.6)		22-24	404 (46.4)	68 (46.9)		34-36	386 (38.4)	98 (39)	
Paternal age (yrs)			0.781	Paternal age (yrs)			0.890	Paternal age (yrs)			0.970
<30	101 (10.5)	22 (11.4)		<30	65 (7.5)	10 (6.9)		<30	102 (10.2)	26 (10.4)	
30-35	678 (70.3)	130 (67.4)		30-35	547 (62.9)	92 (63.4)		30-35	663 (66)	162 (64.5)	
36-40	149 (15.4)	31 (16.1)		36-40	216 (24.8)	34 (23.4)		36-40	177 (17.6)	46 (18.3)	
>40	37 (3.8)	10 (5.2)		>40	42 (4.8)	9 (6.2)		>40	62 (6.2)	17 (6.8)	
Maternal age (yrs)			0.953	Maternal age (yrs)			0.813	Maternal age (yrs)			0.973
<30	296 (30.7)	58 (30.1)		<30	173 (19.9)	27 (18.6)		<30	258 (25.7)	62 (24.7)	
30-35	565 (58.5)	115 (59.6)		30-35	614 (70.6)	101 (69.7)		30-35	621 (61.9)	157 (62.5)	
36-40	97 (10.1)	18 (9.3)		36-40	59 (6.8)	13 (9)		36-40	112 (11.2)	28 (11.2)	
>40	7 (0.7)	2 (1)		>40	24 (2.8)	4 (2.8)		>40	13 (1.3)	4 (1.6)	

Note: All the P value>0.05, indicating no difference of demographic characteristic between the norm population and study population; SP=Study population.

Table S2. Rating criteria based on norms of three age groups.

**Area**	Physiological age (month)			Rating
	1–12 (n = 965)	13–24 (n = 870)	25–36 (n = 1004)	
GM	≥-2	≥-4	≥-7	Qualified
	<-2	<-4	<-7	Delayed
FM	≥-2	≥-5	≥-7	Qualified
	<-2	<-5	<-7	Delayed
CG	≥-1	≥-4	≥-6	Qualified
	<-1	<-4	<-6	Delayed
LG	≥-2	≥-4	≥-7	Qualified
	<-2	<-4	<-7	Delayed
SE	≥-2	≥-5	≥-7	Qualified
	<-2	<-5	<-7	Delayed
SC	≥-2	≥-4	≥-7	Qualified
	<-2	<-4	<-7	Delayed

Note: GM=Gross motor, FM=Fine motor, CG=Cognition, LG=Language, SE=Social emotion, SC=Self-care; the rating criteria was based on the difference between development age and physiological age, for example, if a child aged 6 years had the GM development age of 4 years (4-6≥-2), then he was regarded as "qualified" in GM according to our criteria.
